# Critical periods in adult neurogenesis and possible clinical utilization of new neurons

**DOI:** 10.3389/fnins.2014.00177

**Published:** 2014-06-24

**Authors:** Masahiro Yamaguchi, Kensaku Mori

**Affiliations:** ^1^Department of Physiology, Graduate School of Medicine, The University of TokyoTokyo, Japan; ^2^CREST, Japan Science and Technology AgencySaitama, Japan

**Keywords:** critical period, plasticity, animal age, cellular age, behavioral state, experience-dependent, rehabilitation

## Critical periods in animal age and in cellular age in mammals

In mammals, sensory and motor experiences during the neonatal and infant stages of growth induce remarkable plastic changes in the structural organization and functional properties of neuronal circuits in the brain. Following this critical period, however, experience-dependent plasticity declines significantly. The term “critical period” therefore refers to a specific period in early life during which experience-dependent plasticity is greatly potentiated (Levelt and Hübener, 2012), presumably because neuronal circuits and their constituent young neurons transiently express a high capacity for plasticity.

The presence of adult neurogenesis in the olfactory bulb and dentate gyrus of the hippocampus raises the possibility that the critical period might be extended into adulthood in these particular brain regions. Intriguingly, while ocular dominance plasticity of the visual cortex, a non-neurogenic region in the adult brain, occurs only during a few weeks in the neonatal period in rodents, transplantation of new neurons to the adult visual cortex restores the ocular dominance plasticity (Southwell et al., 2010). The occurrence of adult neurogenesis raises the possibility that the critical period of plasticity can be extended into or restored in adulthood by supplying new neurons with high plastic capacity (Table 1).

**Table 1:** **Time windows for experience-dependent plasticity in adult neurogenesis and cell transplantation therapy**.

**Types of time windows**	**Adult neurogenesis**	**Cell transplantation**
Animal age	Extended plasticity of neuronal circuits into adulthood	Functional restoration and improvement of damaged or aged brain
Cellular age	Developmental stage of adult-born neurons with high experience-dependent plasticity	Developmental stage of transplanted neurons with high rehabilitation-dependent plasticity
Behavioral state	Sequence of experience during wakefulness and subsequent rest or sleep	Sequence of rehabilitation and subsequent post-rehabilitation period

In parallel with the idea of a “critical period in animal age,” we previously noted that adult-born neurons have “critical periods in cellular age,” during which the newly generated neurons are either incorporated into or eliminated from the preexisting host neuronal circuits (Table 1) (Yamaguchi and Mori, 2005). Manifestation of the highly plastic features of adult-born neurons might therefore depend on their cellular age. The idea of a “critical period in cellular age” appears fundamental to the study of adult neurogenesis. The experience-dependent plasticity of neuronal circuits in the olfactory bulb and hippocampus appears to result from interaction between newly generated neurons and preexisting neuronal circuits, the former having higher plasticity and latter having lower plasticity. As a result, the differences observed in the plastic potential of new neurons between that during the critical period and that during the subsequent period are attributable to the different properties of new neurons at different cellular ages. In fact, analysis of adult-born neurons has revealed many aspects of the critical period of experience-dependent plasticity, including the presence of critical periods for the survival or death decision, for synapse formation, for synaptic plasticity, and for incorporation into functional neuronal circuits (Yamaguchi and Mori, 2005; Ge et al., 2007; Kee et al., 2007; Tashiro et al., 2007; Kelsch et al., 2009; Nissant et al., 2009; Belnoue et al., 2011).

A second important issue in adult neurogenesis is the time of day at which the plastic change in neuronal circuits occurs. We previously found that the life and death decision of new neurons in the olfactory bulb occurs within a narrow time window, in association with a specific behavioral state of the animal (Yokoyama et al., 2011). In food-restricted mice, extensive apoptotic elimination of new olfactory bulb neurons occurs during several tens of minutes of rest or sleep after food eating. The extent of neuronal elimination during postprandial rest or sleep is influenced by olfactory sensory experience. Thus, the life and death of new olfactory bulb neurons is likely determined within a particular “behavioral state-correlated time window”, which consists of the sequence of olfactory behavior during wakefulness and subsequent rest or sleep behavior (Table 1). The behavioral state-correlated time window may be further interpreted as a “critical time window of preexisting neuronal circuits” that determines whether to incorporate or eliminate new neurons.

## Contribution of critical period analysis of adult neurogenesis to brain repair by cell transplantation

We consider that the “critical period of adult-born neurons” and “behavioral state-correlated time window for plasticity” ideas have important implications for the possible clinical utilization of new neurons. To supplement new neurons to diseased or injured brains, neural stem/progenitor cells are the plausible candidate for clinical use. Neural stem/precursor cells from various sources can be utilized, including those from embryonic brains, those induced from ES cells or iPS cells, and those endogenously present in the patient's brain (Bellenchi et al., 2013; Sandoe and Eggan, 2013). Whatever the origins of stem/precursor cells, it is assumed that transplantation or recruitment of stem/precursor cells into damaged neuronal circuits might restore the function of the neuronal circuits via the process of their development into mature neurons and integration into the preexisting neuronal circuits. Rehabilitation following brain damage is critically aided by physical exercise, intellectual exercise and extensive sensory stimulation. In stem/progenitor cell transplantation, a primary aim of rehabilitation would be to provide proper experience-dependent activity to transplanted cells and recipient neuronal circuits, with the expectation that the transplanted cells develop and survive and are appropriately incorporated into the recipient neuronal circuits for the recovery of circuit function. On this basis, knowledge of the critical period of experience-dependent plasticity of adult-born neurons will provide a scientific basis for cell transplantation-based rehabilitation therapy (Döbrössy et al., 2010).

Application of our understanding of the critical period in the cellular age of adult-born neurons provides insight into how the timing of rehabilitation and cell transplantation is coordinated (Table 1). Adult neurogenesis studies suggest that critical periods of experience-dependent survival, synapse formation, synaptic plasticity and circuit incorporation of adult-born neurons correspond to their synaptogenesis or post-synaptogenesis period (Kelsch et al., 2010; Toni and Sultan, 2011; Drew et al., 2013; Lepousez et al., 2013). By analogy, rehabilitation during the period when transplanted neurons make synaptic contacts with preexisting neurons might crucially regulate the appropriate incorporation of transplanted neurons into recipient neuronal circuits.

Knowledge of the “behavioral state-correlated time window for plasticity” also indicates the possibility that the occurrence of specific behavioral states after rehabilitation might be crucial (Table 1). In the hippocampus and neocortex, neuronal activity during rest or sleep is considered to reflect neuronal activity during the preceding waking period (Diekelmann and Born, 2010). The life and death decision of adult-born olfactory bulb neurons might be promoted by neuronal activity during rest or sleep which reflects the neuronal activity during the preceding waking period with olfactory experience (Yamaguchi et al., 2013). If we assume that this “olfactory experience” period during wakefulness corresponds to the rehabilitation period in cell transplantation therapy, the fate decision of transplanted neurons about whether they will be incorporated or eliminated does not occur during rehabilitation, but may actually be promoted during the post-rehabilitation period. If this is the case, significant attention should be paid as to how patients spend time after rehabilitation. How long is the effect of rehabilitation maintained until the following period of neuronal selection? Is it better to rest or sleep immediately after rehabilitation? Is the effect of rehabilitation perturbed or erased by other unrelated experiences before rest or sleep? These questions cannot now be answered with any reliability, but consideration to the possible role of post-rehabilitation period will likely optimize the effect of rehabilitation.

Further, applying the idea that the critical period of plasticity can be extended or restored by supplying new neurons suggests that brain repair by cell transplantation may allow not only the recovery of brain function, but also the improvement of function. Use of the highly plastic potential of transplanted neurons might allow an improvement in brain function beyond its original pre-pathological/trauma level, which cannot be achieved solely by preexisting old neurons (Table 1).

## How far are we from the clinical utilization of basic knowledge gathered from critical period studies in adult neurogenesis?

As discussed, the basic idea of critical periods in the study of adult neurogenesis is applicable to cell transplantation therapy. However, the actual time window for experience-dependent plasticity is unlikely to be uniform. Critical periods in cellular age would depend not only on the type of transplanted neurons but also on the type and condition of recipient neuronal circuits (recipient brain regions). The coordinated timing of rehabilitation after cell transplantation needs to be identified in individual cases. The difficulties of studying the human brain *in vivo* require extensive study in experimental animals and a way to extrapolate the results back to humans.

At present, little is known about the molecular mechanisms underlying the critical periods of plasticity. Knowledge about the expression and function of neurotransmitter receptors, including NMDA, AMPA, and GABA receptors, during the development of adult-born neurons is accumulating, and signals via these receptors have been found to be crucial to the survival and synapse formation of adult-born neurons (Kelsch et al., 2010; Toni and Sultan, 2011; Drew et al., 2013; Lepousez et al., 2013). However, the developmental relationship between the expression of these receptors and the opening and closing of the critical periods of plasticity is unclear, and the molecular mechanisms underlying the behavioral state-correlated time window for plasticity are not understood at all. Further detailed understanding of the mechanisms of these critical time windows will enable the development of pharmacological and molecular interventions to better coordinate the rehabilitation to cell transplantation therapy.

A further critical point is that while most studies of adult neurogenesis involve healthy brains, cell transplantation therapy is primarily conducted in aged or damaged brains. Recipient neuronal circuits in cell transplantation therapy are unlikely to provide an ideal environment and experience-dependent activity to new neurons. To best utilize our understanding of adult neurogenesis and optimize outcomes from cell transplantation, the recipient brain must be brought to a healthy state as possible. Cell transplantation therapy needs to be combined with the understanding of the nature of the disease/injury of the recipient brain and the application of promising interventions to it (Gerin et al., 2011; Gandy and DeKosky, 2013). Rehabilitation before cell transplantation (Döbrössy and Dunnett, 2005) might have positive effect on the outcomes. We need to know more about the interaction between new neurons and preexisting neuronal circuits in the appropriate utilization of new neurons, and to understand the effect of rehabilitation and pharmacological/molecular interventions on both new neurons and recipient neuronal circuits.

Rehabilitation depends on the active participation of patients, and requires the cooperation of patients, doctors and paramedical staff. A scientific understanding of rehabilitation in relation to the critical time windows of experience-dependent plasticity would boost motivation for rehabilitation, and enable efforts to be concentrated on particular time windows. Research into the critical time windows of plasticity in adult-born neurons will contribute to the development of effective rehabilitation programs, and support patients in their hopes and motivation to recover from brain damage.

### Conflict of interest statement

The authors declare that the research was conducted in the absence of any commercial or financial relationships that could be construed as a potential conflict of interest.
